# Knock-Down of Mucolipin 1 Channel Promotes Tumor Progression and Invasion in Human Glioblastoma Cell Lines

**DOI:** 10.3389/fonc.2021.578928

**Published:** 2021-04-19

**Authors:** Giorgio Santoni, Consuelo Amantini, Massimo Nabissi, Federica Maggi, Antonietta Arcella, Oliviero Marinelli, Anna Maria Eleuteri, Matteo Santoni, Maria Beatrice Morelli

**Affiliations:** ^1^Immunopathology Laboratory, School of Pharmacy, University of Camerino, Camerino, Italy; ^2^Immunopathology Laboratory, School of Biosciences and Veterinary Medicine, University of Camerino, Camerino, Italy; ^3^Department of Molecular Medicine, Sapienza University, Rome, Italy; ^4^Neuropathology Laboratory, Istituto di Ricovero e Cura a Carattere Scientifico Neuromed, Pozzilli, Italy; ^5^Clinical Biochemistry Laboratory, School of Biosciences and Veterinary Medicine, University of Camerino, Camerino, Italy; ^6^Medical Oncology Unit, Hospital of Macerata, Macerata, Italy

**Keywords:** mucolipin, TRPML1, invasion, proliferation, senescence, apoptosis, autophagy, cathepsin B

## Abstract

Among cancers that affect the central nervous system, glioblastoma is the most common. Given the negative prognostic significance of transient receptor potential mucolipin 1 (TRPML1) channel reduction in patients with glioblastoma, as discussed in previous publications, the aim of the current study was to investigate the biological advantage of TRPML1 loss for glioma cells. Human glioblastoma primary cancer cells (FSL and FCL) and glioblastoma cell lines (T98 and U251) were used for that purpose. TRPML1 silencing in T98 cells induces defective autophagy, nitric oxide (NO) production, and cathepsin B-dependent apoptosis in the first 48 h and then apoptotic-resistant cells proliferate with a high growth rate with respect to control cells. In U251 cells, knock-down of TRPML1 stimulates NO generation and protein oxidation, arrests cell cycle at G2/M phase, and induces autophagy leading to cathepsin B-dependent senescence. Finally, in both cell lines, the long-term effects of TRPML1 silencing promote survival and invasion capacity with respect to control cells. Silencing of TRPML1 also affects the phenotype of glioblastoma primary cells. FSL cells show increased proliferation ability, while FCL cells enter into senescence associated with an increased invasion ability. In conclusion, although the molecular heterogeneity among different glioblastoma cell lines mirrors the intercellular heterogeneity in cancer cells, our data support TRPML1 downregulation as a negative prognostic factor in glioblastoma.

## Introduction

Glioblastoma is a malignant tumor of the brain (1), accounting for more than half of all astrocytoma cases (2). Classified high-grade gliomas have a poor prognosis and are characterized by proliferation, necrosis, angiogenesis, invasion, and evasion of apoptosis (3). Despite attempts to undertake molecular stratification of patients with glioma in order to achieve the most suitable therapeutic strategies, the median survival of patients with glioblastoma is 12–18 months (4). These lethality aspects could involve high therapeutic resistance together with a high capacity of migration/invasion that make it impossible to augment the aggressiveness of this tumor with complete surgical resection (5). Furthermore, a number of studies have identified the presence of multiple molecular subtypes within an individual tumor and also multiple subtypes when the tissue was obtained from spatially distinct regions within an individual patient demonstrating a significant intra- and inter-tumoral heterogeneity in glioblastoma (6, 7). This particular characteristic of glioblastoma is closely connected with its resistance to conventional treatment and its recurrence; indeed, it has been demonstrated that heterogeneity accumulates during glioma cell invasion (8).

Several findings suggest that endolysosomal cation channels are involved in proliferation, angiogenesis, and metastasis in different types of cancer (9, 10). Inside the group of endolysosomal nonselective cation channels, the transient receptor potential mucolipin 1 (TRPML1) is one of the major actors in calcium-release. It belongs to the TRP family, and it is characterized by six-transmembrane-spanning domains that contain a pore-lining domain, a ligand/voltage-sensing domain, and a large extracellular loop between transmembrane segments 1 and 2 (11). TRPML1 is encoded by the *MCOLN1* gene and activated by the phosphatidylinositol-3,5-biphosphate [PtdIns(3,5)P2], one of the major components of endolysosomal membranes (12–16). Moreover, TRPML1 has an intraluminal loop whose protonation stimulates channel activation (17–19), and it is inhibited by phosphatidylinositol-4,5-biphosphate [PtdIns(4,5)P2], sphingomyelins, and lysosomal adenosine (15, 16). Given that TRPML1 promotes the cation efflux into the cytosol (20), its activity is involved in membrane-trafficking processes, autophagic vesicle (AV)–lysosome fusion, lysosome reformation, lysosomal exocytosis, and pH homeostasis (21). TRPML1 mutations are responsible for the Mucolipidosis type IV (MLIV), an autosomal recessive lysosomal storage disease with a peculiar phenotype (psychomotor alterations, corneal opacities, and achlorhydria) (22). MLIV fibroblasts present defects in macroautophagy with a resulting abnormal accumulation of ubiquitinated protein inclusions that contribute to the neurodegenerative phenotype (23–25).

TRPML1 plays a role in the homeostatic control of steady state of cells. In normal cells, growth factor or glucose deprivation and mild oxidative stress triggers the macroautophagy or the chaperone-mediated autophagy that rescues the stress-exposed cells from death, whereas severe oxidative stress stimulates autophagic cell death or apoptosis (26). In addition, the activation of TRPML1 by specific agonists induces cytotoxic effects (27). Increased intracellular reactive oxygen species (ROS) levels directly activate TRPML1 inducing lysosomal Ca^2+^ release, this triggers PPP3/calcineurin-dependent transcription factor EB (TFEB)nuclear translocation prompting autophagy (28, 29). It is interesting to notice that TRPML1 and TFEB take part in a feedback loop whereby TRPML1 controls TFEB activity and constitutes both a downstream transcriptional target and a major effector of TFEB biological activity (28, 29). Autophagy represents the front line of defense against oxidative stress not only in normal but also in neoplastic cells (30).

Several papers have demonstrated TRPML1 to be associated with the acquisition of tumor phenotype (31–36). In HRAS-driven cancer cells, increased TRPML1 activity is required for the plasma membrane localization of cholesterol (35). Indeed, a mislocalization of cholesterol from the plasma membrane to the endolysosomes is induced by knock-down of *MCOLN1 or* inhibition of TRPML1 leading to the suppression of extracellular-signal-regulated kinase (ERK) phosphorylation and cell proliferation (35). TRPML1-deficient melanoma cells exhibit decreased survival, proliferation, tumor growth, macropinocytosis, and proteotoxic stress (36). A high level of TRPML1 expression was associated with poor clinical characteristics in patients with pancreatic ductal adenocarcinoma. The silencing of *MCOLN1* blocks the proliferation of PDAC cells (31). Triple-negative breast cancer (TNBC) expresses high TRPML1 levels; its downregulation suppresses cancer growth (37). The TRPML1 expression is decreased in human non-small cell lung cancer (NSCLC) tissues compared to normal ones, but it increases in advanced stages, suggesting that TRPML1 may confer a survival advantage (32). Indeed, the inhibition of TRPML1 suppressed the proliferation, migration, and invasion and the NSCLC cells, whereas its overexpression promotes autophagy in the A549 and H1299 cells, keeping the metabolism and energy requirements of the tumor in balance (32).

Unlike the abovementioned studies, downregulation of TRPML1 expression compared to normal human astrocytes has been reported in glioblastoma (33). Furthermore, the loss or reduction of TRPML1 expression was correlated with short survival in patients with glioblastoma, suggesting that it may represent a negative prognostic factor (33). However, to date, the mechanisms remain unknown.

The present study examined the biological effect of TRPML1 knock-down through an *in vitro* study of the commonly used human glioblastoma cell line models, T98 and U251 along with human glioblastoma-derived primary cancer cells.

## Materials and Methods

### Cell Lines

The glioblastoma T98 and U251 cell lines (grade IV), obtained from the European Collection of Cell Cultures (ECACC, Salisbury, UK), were maintained in Eagle's minimum essential medium (EMEM, Lonza Bioresearch, Basel, Switzerland) supplemented with 10% heat-inactivated fetal bovine serum (FBS), 2 mmol/L L-glutamine, 100 IU/ml penicillin, and 100 μg streptomycin. The primary glioblastoma cell lines, FSL and FCL, were prepared from enzymatic digestion of the bioptic sample, surgically removed from patients with grade IV glioblastoma, who gave informed consent to the study and isolated as described previously (38). FSL and FCL were cultured in Dulbecco's modified Eagle's medium (DMEM; Lonza Bioresearch, Basel, Switzerland) supplemented with 10% FBS, 2 mmol/L L-glutamine, 100 IU/ml penicillin, and 100 μg streptomycin. All cell lines were maintained at 37°C, 5% CO_2_, and 95% humidity.

### Chemical and Reagents

The 3-(4,5-dimethylthiazol-2-yl)-2,5-diphenyltetrazolium bromide (MTT), propidium iodide (PI, 2 μg/ml), ribonuclease A (100 μg/ml), dichlorodihydrofluorescein diacetate (DCFDA, 20 μM), bromodeoxyuridine (BrdU), and CA074Me (**c**athepsin B inhibitor, 2.5 μM) were purchased from Sigma-Aldrich (Milan, Italy). Bafilomycin A1 (BAF A1, 25 nM) was from Invitrogen (Toulouse, France). Annexin V-fluorescein isothiocyanate (FITC) from Enzo Life Sciences (Farmingdale, NY, USA). 4-Amino-5-methylamino-2′, 7′-difluorofluorescein diacetate (DAF-FM) was purchased from Abcam (Cambridge, UK). The following rabbit polyclonal antibodies (Abs) were used: anti-microtubule-associated protein-1 light chain 3 (LC3, 2 μg/ml, Novus Biologicals, Littleton, CO, USA), anti-p62 (1:1,000, Cell Signaling Technology, Danvers, MA, USA), anti-cathepsin B (250 ng/ml, Calbiochem, San Diego, CA, USA), anti-pERK (1:1,000, Cell Signaling Technology, Danvers, MA, USA), anti-ERK (1:1,000, Cell Signaling Technology, Danvers, MA, USA), anti-pAKT (1:1,000, Cell Signaling Technology, Danvers, MA, USA), and anti-AKT (1:1,000, Cell Signaling Technology, Danvers, MA, USA). The following mouse monoclonal Abs were used: anti-TRPML1 (1:300 for Western blot, Santa Cruz Biotechnology, Dallas, TX, USA), anti-LAMP1 (1:1,000, Santa Cruz Biotechnology, Dallas, TX, USA), anti-β-actin (1:1,000, Santa Cruz Biotechnology, Dallas, TX, USA), and anti-glyceraldehyde-3-phosphate dehydrogenase (anti-GAPDH, 14C10, 1:1,000, Cell Signaling Technology, Danvers, MA, USA). The following secondary antibodies were used: horseradish peroxidase (HRP)-conjugated anti-mouse IgG and HRP-conjugated anti-rabbit IgG (1:2,000, Cell Signaling Technology, Danvers, MA, USA).

### Difluorofluorescein Diacetate and DCFDA Staining

To quantify the intracellular nitric oxide (NO) and ROS levels, DAF-FM diacetate and DCFDA-based assay were used, respectively. Cells were stained with 10 μM of DAF-FM or 20 μM of DCFDA for 45 min at 37°C and 5% CO_2_. The cells were then washed, and the intensity of the fluorescence was assayed using the flow cytometry and CellQuest software (BD Biosciences, San Jose, CA, USA).

### Oxidized Protein Analysis

An OxyBlot Protein Oxidation Detection Kit (Merck Millipore, Burlington, MA, USA) was used to detect The Oxidized proteins, according to the instructions of the manufacturer. Dinitrophenyl hydrazine was added to crude total proteins (20 μg) to derive carbonyl groups from the protein side chains. Carbonylated proteins were resolved on sodium dodecyl sulfate-polyacrylamide gel electrophoresis, and the Western blot analysis was performed using the provided anti-DNP antibody (1:150). The detection was performed using the LiteAblot PLUS or Turbo Kits (EuroClone, Milan, Italy), and DNP signals were quantified by densitometry using the Quantity One software (version 4.6.7, Bio-Rad, Hercules, CA, USA).

### Western Blot Analysis

Cells were lysed in a lysis-buffer containing protease inhibitor cocktail (EuroClone, Milan, Italy). Proteins were separated on 8–14% SDS polyacrylamide gel in a Mini-PROTEAN Tetra Cell System (Bio-Rad, Hercules, CA, USA). Protein transfer was carried out using a Mini Trans-Blot Turbo RTA System (Bio-Rad, Hercules, CA, USA). Membranes previously blocked with 5% low-fat dry milk and 2% bovine serum albumin (BSA) in phosphate-buffered saline 0.1% Tween 20 were incubated overnight at 4°C in primary Abs (anti-TRPML1, anti-LC3, anti-p62, anti-cathepsin B, anti-LAMP1, anti-pERK, anti-ERK, anti-pAKT, anti-AKT, anti-β-actin, and anti-GAPDH), followed by the incubation for 1 h at room temperature with HRP-conjugated anti-rabbit or anti-mouse secondary Abs. The detection was performed using the LiteAblot PLUS or Turbo Kits (EuroClone, Milan, Italy), and densitometric analysis by a Chemidoc using the Quantity One software (version 4.6.7, Bio-Rad, Hercules, CA, USA). GAPDH and β-actin were used as loading controls. One representative out of three independent experiments was shown in each immunoblot figure.

### Cathepsin B and Caspase 3 Activity

The cathepsin B proteolytic activity was measured using the fluorogenic peptide Z-Arg-Arg-AMC at a final concentration of 50 μM. The cathepsin D activity was analyzed to verify the assay and was used as negative control. The mixture, containing 5 μg of cell lysate, was preincubated in 100 mM phosphate buffer pH 6.0, 1 mM EDTA, and 2 mM dithiothreitol for 5 min at 30°C. Upon the addition of the substrate, the mixture was incubated for 15 min at 30°C. The caspase three activity was measured with the fluorogenic substrate Ac-Asp-Glu-Val-Asp-AMC (5 μM final concentration) and 10 μg of cell lysate in 50 mM Tris/HCl, 50 mM NaCl, 5 mMCaCl_2_, 1 mM EDTA, 0.1% CHAPS, 5 mM β-mercaptoethanol, pH 7.5. Incubation was for 60 min at 37°C. The fluorescence of hydrolyzed AMC was measured on a SpectraMax Gemini XPS Microplate Reader (λ_exc_ = 365 nm and λ_em_ = 449 nm; Molecular Devices, San Jose, CA, USA). The effective cathepsin B and caspase three contribution to the proteolysis was evaluated through control experiments performed using the specific inhibitors Ca074Me and Z-D(OMe)-E(OMe)-V-D(OMe)-FMK (DEVD-FMK), respectively, and subtracting these values from the fluorescence values obtained by analyzing cell lysates.

### Transient Receptor Potential Mucolipin 1 Knock-Down Model

Transient receptor potential mucolipin 1 (siTRPML1) and siCONTROL non-targeting siRNA (siGLO, used as negative control) FlexiTube siRNA were purchased from QIAGEN (Milan, Italy). T98, U251, FSL, and FCL cell lines were plated at the density of 1.2 × 10^5^/ml, and siTRPML1 or siGLO (150 ng) was added to the wells, following the HiPerfect Transfection Reagent protocol (QIAGEN, Milan, Italy). No differences were observed comparing siGLO transfected with untransfected cells.

### Cell Growth Assay

About 3 × 10^4^/ml untreated, siGLO or siTRPML1 glioma cells were plated in 96-well plates up to 72 h. Then, 0.8 mg/ml of MTT was added to the samples and incubated for an additional 3 h. After the removal of the medium from the wells, the formazan crystals were dissolved in 100 μl per well of dimethyl sulfoxide (DMSO), and the colored solutions were read by microtiter plate spectrophotometer (BioTek Instruments, Winooski, VT, USA). Four replicates were used for each treatment.

### Cell Cycle Analysis

Cells were fixed in ice-cold 70% ethanol, treated for 30 min at 37°C with 100 μg/ml ribonuclease A solution, stained for 30 min at room temperature with PI 20 μg/ml, and analyzed at 48 h after transfection by flow cytometry using linear amplification.

### Cell Proliferation Assay

Cell proliferation was assessed by BrdU-ELISA using a BrdU Cell Proliferation ELISA Kit (Abcam, Cambridge, UK) according to the protocol of the manufacturer. Data were quantified using a spectrophotometer set at a dual wavelength of 450/550 nm.

### Senescence Analysis

At 24, 48, and 72 h after transfection, cells were incubated for 1 h at 37°C and 5% CO_2_ with 100 nM BAF A1 in culture medium to induce lysosomal alkalinization at pH 6 followed by 1-h incubation with 33 μM C_12_FDG. Samples were immediately analyzed using the FACScan cytofluorimeter and the CellQuest software (BD Biosciences, San Jose, CA, USA). The C12-fluorescein signal was measured on the FL-1 detector, and β-galactosidase activity was estimated as a percentage of positive cells.

### Cell Death Analysis

Cell death was evaluated at different times (24, 48, and 72 h) using the FITC-conjugated Annexin V staining. Briefly, treated cells were incubated with 5 μl Annexin V-FITC for 10 min at room temperature. The cells were then washed, and the intensity of the fluorescence was assayed using the flow cytometry and CellQuest software (BD Biosciences, San Jose, CA, USA).

### Invasion Assay

Cell invasion was evaluated by Transwell assay using the Transwell Chambers (BD Biosciences, Franklin Lakes, NJ, USA) characterized by the upper face of the polycarbonate membrane in the upper chamber covered with 1 mg/ml Matrigel (BD Biosciences, San Jose, CA, USA). A total of 750 μl cell culture medium supplemented with 10% FBS was added in the lower chamber. A serum-free culture medium (500 μl) containing 2.5 × 10^4^ siTRPML1 or siGLO glioma cells was plated into the upper chamber. After 24 h of incubation, Matrigel (BD Biosciences, San Jose, CA, USA) and the cells remaining in the upper chamber were removed by cotton swabs. The cells on the lower surface of the membrane were fixed in 4% paraformaldehyde, stained with DAPI, and observed using a fluorescence microscope (BX51 Fluorescence Microscope, Olympus, Milan, Italy). A total of 10 fields at ×10 magnification were selected at random to measure the average cell coverage using the ImageJ software version 1.45s (National Institutes of Health, Bethesda, MD, USA). The experiments were performed in triplicate at least three times independently.

### Statistical Analysis

The statistical significance was determined by the Student's *t*-test and by one-way ANOVA, followed by Bonferroni's posttest. No statistically significant difference was found between untransfected and siGLO transfected U251 and T98 cells (data not shown).

## Results

### Transient Receptor Potential Mucolipin 1 Silencing Affects Glioblastoma Cell Lines Viability

**Figure 1:**
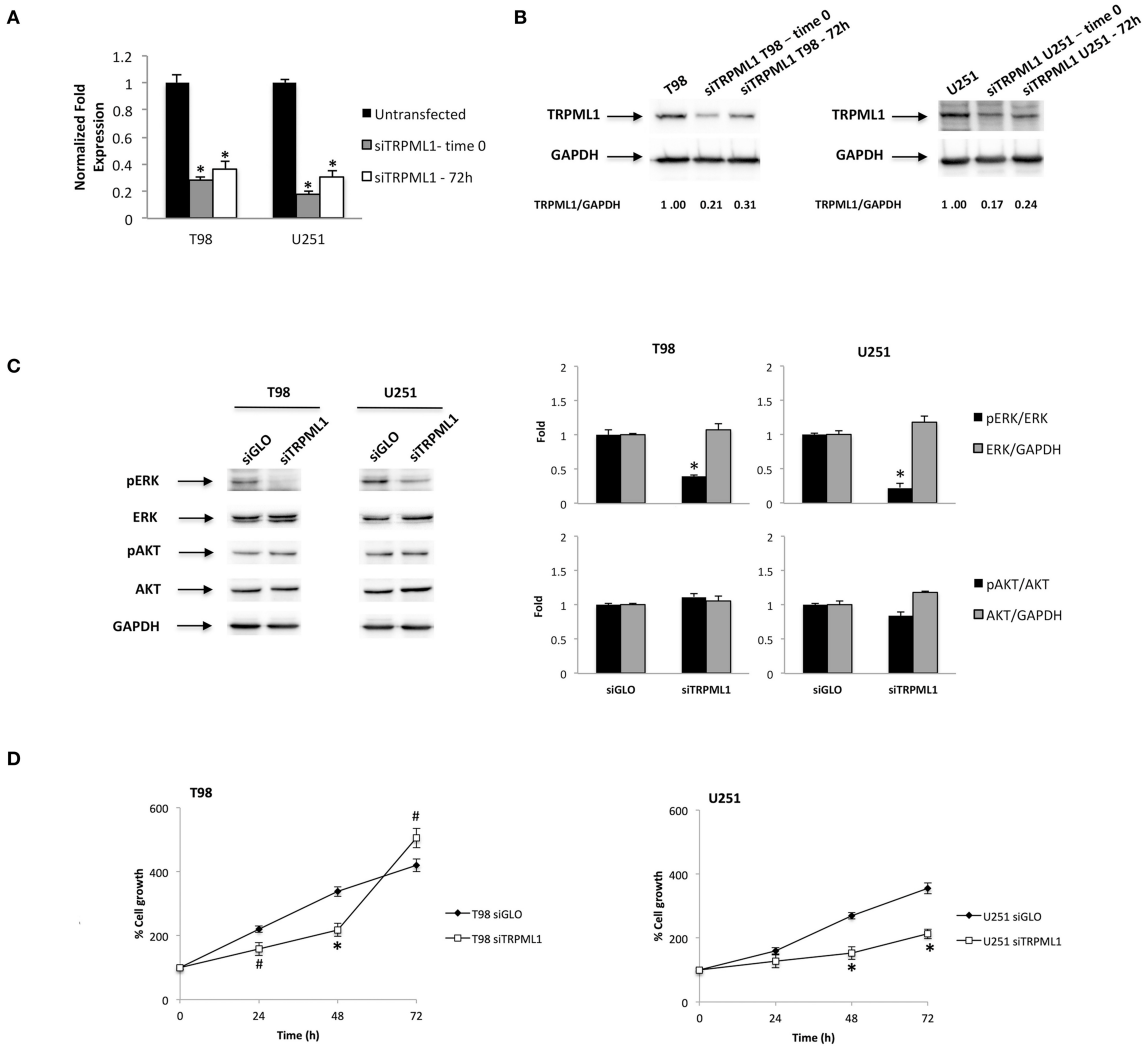
Transient receptor potential mucolipin 1 (TRPML1) silencing affects glioblastoma cell viability. **(A)** The relative TRPML1 mRNA expression in untransfected and siTRPML1 T98 and U251 glioma cell lines after 72 h of silencing (time 0) and after 72-h post-transfection was evaluated by the quantitative reverse transcription (qRT)-PCR. TRPML1 mRNA levels were normalized for glyceraldehyde-3-phosphate dehydrogenase (GAPDH) expression. Data are expressed as mean ± SD; *p < 0.001 vs. untransfected. **(B)** The Western blot analysis of TRPML1 protein levels. Blots are representative of one of three separate experiments. TRPML1 densitometry values were normalized to GAPDH used as loading control. The TRPML1 protein levels of silenced samples were determined with respect to TRPML1 levels of untransfected cells. **(C)** The Western blot analysis of pERK, ERK, pAKT, and AKT protein levels in siGLO and siTRPML1 T98 and U251 cells after 72 h of silencing (time 0). Blots are representative of one of three separate experiments. ERK and AKT densitometry values were normalized to GAPDH used as loading control. The pERK and pAKT protein levels were determined with respect to ERK and AKT levels. *p < 0.01 siTRPML1 vs. siGLO. **(D)** Cell viability was evaluated by 3-(4,5-dimethylthiazol-2-yl)-2,5-diphenyltetrazolium bromide (MTT) assay in siGLO control and in siTRPML1 T98 and U251 glioblastoma cells for up to 72 h. Data shown are expressed as mean ± SE of three separate experiments. *p < 0.01 and ^#^*p* < 0.05 siTRPML1 vs. siGLO.

We previously investigated the expression of TRPML1 in 66 samples of human glioblastoma tissues (33). Given that the reduction or loss of the TRPML1 mRNA expression strongly correlated with short survival, we suggested that the reduction of the TRPML1 expression could represent a negative prognostic factor in patients with glioblastoma. We are now aiming to study *in vitro* the possible advantages of the TRPML1 downregulation in glioblastoma cells, exploiting small interfering RNA (siRNA) against TRPML1 whose specificity has been previously verified (33). We evaluated the gene silencing efficacy in T98 and U251 cell lines by the quantitative reverse transcription (qRT)-PCR and Western blot analysis. TRPML1 mRNA levels were decreased by about 80% in T98 and U251 cells silenced for TRPML1 (siTRPML1) after 72 h of transfection that we called time 0, and the silencing was maintained up to 72-h post-transfection as shown in Figure 1A. The reduced expression of TRPML1 was confirmed at protein level by the Western blot analysis (Figure 1B).

As the first step, we characterized pERK and pAKT levels related to silenced TRPML1 on both glioma cell lines at time 0 by Western blot. The transfected models result in a decreased ERK activation calculated as pERK/ERK ratio (Figure 1C). Instead, no significant changes related to AKT activation have been detected. Thus, we evaluated the effects of TRPML1 silencing on T98 and U251 cell growth up to 72-h post-transfection by the MTT assays. Both siTRPML1 T98 and U251 cells showed a reduced viability compared to the transfection control cells (siGLO) until 48 h (Figure 1D). However, increased cell growth was evidenced at 72-h post-transfection in siTRPML1 T98 cells, compared to control (Figure 1D).

### Transient Receptor Potential Mucolipin 1 Knock-Down Results in Oxidative Stress in T98 and U251 Glioma Cells

**Figure 2:**
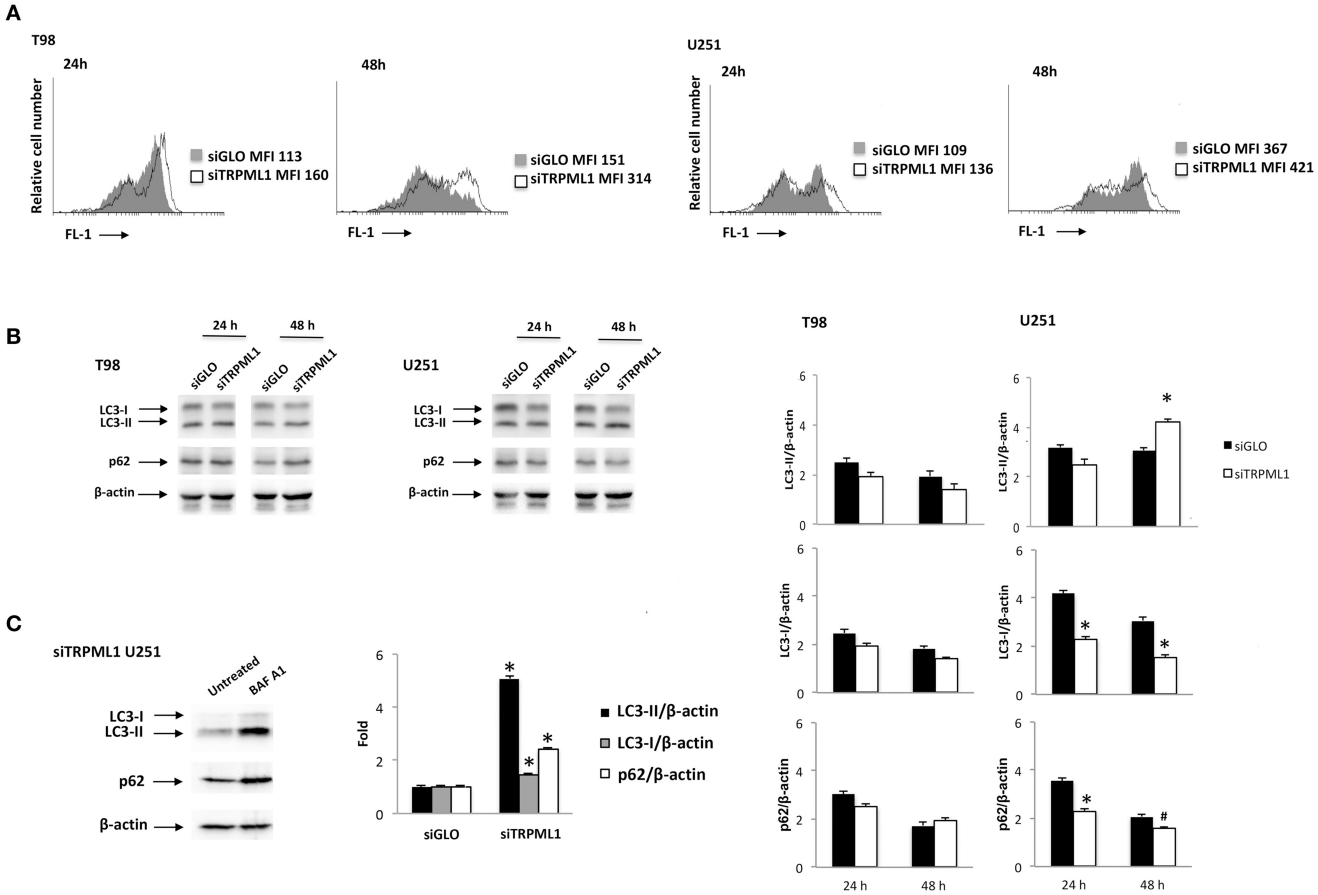
Transient receptor potential mucolipin 1 (TRPML1) silencing induces cellular stress. **(A)** To detect intracellular NO production, cells were stained with DAF-FM before the flow cytometric analysis. Data are expressed as a percentage of dichlorodihydrofluorescein diacetate (DCFDA) positive cells with respect to siGLO control cells. **(B)** The Western blot analysis of LC3 and p62 protein levels in siGLO and siTRPML1 T98 and U251 cell lines after 24- and 48-h post-transfection. Blots are representative of one of three separate experiments. Densitometry values were normalized to β-actin used as loading control. Data of siTRPML1 vs. siGLO cells are expressed as mean ± SE of three separate experiments. *p < 0.01, ^#^*p* < 0.05, siTRPML1 vs. siGLO. **(C)** The Western blot analysis of LC3 and p62 protein levels in siTRPML1 U251 cells untreated or treated for 24 h with BAF A1 25 nM. Blots are representative of one of three separate experiments. Densitometry values were normalized to β-actin used as loading control. Data of BAF A1-treated vs. untreated siTRPML1 U251 cells are expressed as mean ± SE of three separate experiments. *p < 0.01.

Transient receptor potential mucolipin 1 is considered a sensor of oxidative stress because increased ROS levels are able to activate TRPML1-dependent autophagy and lysosome biogenesis (26). Then, silencing of TRPML1 blocks the removal of ROS increasing cellular stress in a model of TRPML1-transfected HeLa cells (39). To characterize the effects of TRPML1 knock-down on glioblastoma cell lines, the production of ROS and nitrogen species (RNS) in siTRPML1 and siGLO T98 and U251 cells were analyzed. No ROS generation was evidenced in siTRPML1 with respect to siGLO T98 and U251 cells (Supplementary Figure 1), whereas an increase in RNS production was observed, as evidenced by the rightward shift in the DAF-FM staining (Figure 2A). The highest RNS levels were evidenced at 48-h post-silencing, in both siTRPML1 cell lines compared to control cells. In particular, T98 cells were more active in RNS production (two-fold) than U251 cells.

A growing amount of evidence links oxidative stress with the autophagy process (40). Therefore, the activation of autophagy was evaluated in siTRPML1 and siGLO T98 and U251 cells at different times post-transfection. Cell lysates were probed with anti-LC3 and anti-p62 Abs and analyzed by Western blot. Increased conversion of the LC3-I in the LC3-II lipidated form and reduced p62 levels, signs of autophagic activity, was identified in siTRPML1 U251 cells, compared to siGLO control cells (Figure 2B). No significative changes in LC3-II and p62 levels were evidenced in siTRPML1 compared to siGLO in T98 cells (Figure 2B). To further confirm the autophagy induction in the U251 cell line, siTRPML1 cells were treated for 24 h with the autophagic inhibitor, Bafilomycin A1 (BAF A1) at 25 nM, and autophagic flux was evaluated. As shown by immunoblots, BAF A1 increased the LC3-II and p62 levels supporting the activation of the autophagic flux in siTRPML1 U251 cells (Figure 2C). Overall, knock-down of TRPML1 induced autophagic survival related to mild oxidative stress in U251 cells, whereas the defective autophagy was evidenced in siTRPML1 T98 cells.

### Effect of TRPML1 Silencing on Cell Proliferation

**Figure 3:**
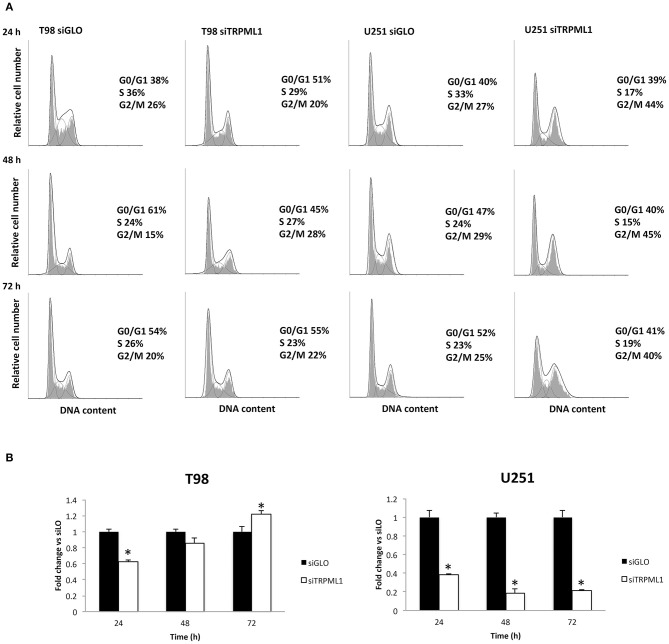
Influence of transient receptor potential mucolipin 1 (TRPML1) silencing on cell proliferation. **(A)** Representative cell cycle distribution in siGLO and siTRPML1 T98 and U251 cell lines at 24-, 48-, and 72-h post-transfection. **(B)** Fold change difference in cell proliferation of siTRPML1 cells was compared with siGLO control cells. *p < 0.01 vs. siGLO.

Autophagy-dependent G2/M cell cycle arrest was reported by Ma et al. on intracellular free radical generation in esophageal cancer cells (41). Thus, cell cycle was evaluated at 24, 48, and 72-h post-transfection in siTRPML1 T98 and U251 cells compared to siGLO control cells (Figure 3A). Knock-down of TRPML1 in U251 cells induced an accumulation of cells in the G2/M phase at all times analyzed. Instead, in siTRPML1 T98 cells, there are no statistically significant differences compared to siGLO cells. We also assessed the effects of TRPML1 silencing on T98 and U251 cell proliferation by the BrdU uptake assays. siTRPML1 U251 cells showed a marked decrease in BrdU incorporation up to 72-h post-transfection compared to siGLO control cells (Figure 3B), supporting data described above. Conversely, siTRPML1 T98 cells exhibited a decrease after 24 h and an enhanced BrdU incorporation at 72-h post-transfection (Figure 3B). Thus, results demonstrated a sustained reduction in siTRPML1 U251 cells growth and a delayed increased proliferation in siTRPML1 T98 cells at 72-h post-transfection.

### The siTRPML1 T98 Cells Undergo Apoptotic Cell Death

**Figure 4:**
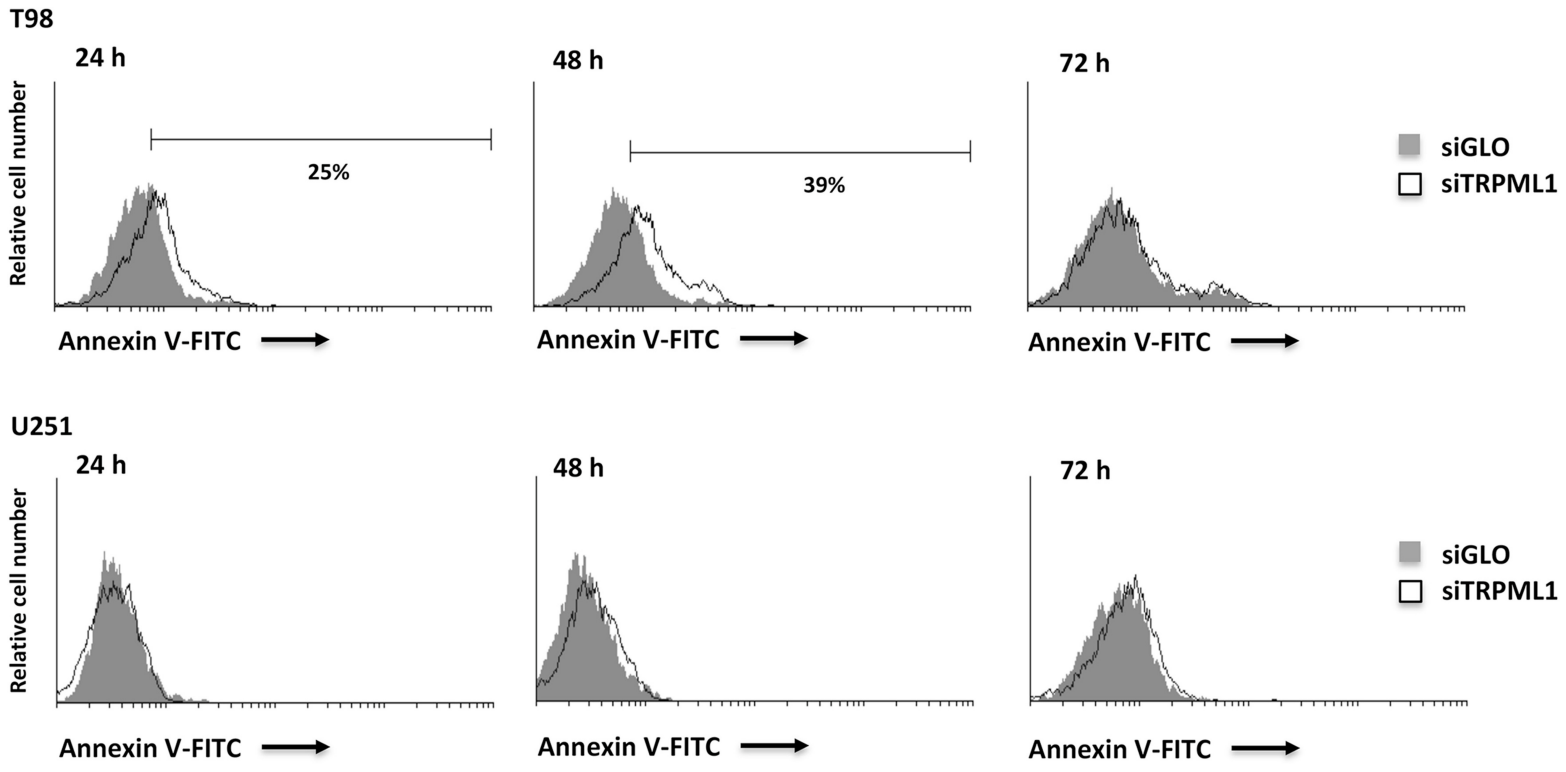
Silencing of transient receptor potential mucolipin 1 (TRPML1) induces apoptotic cell death in T98 cells. Annexin V staining was analyzed by flow cytometry in siGLO and siTRPML1 T98 and U251 cells for up to 72 h of silencing. Percentages represent the positive cells.

Data from the literature suggest that when oxidative stress levels are high, ER functions are impaired, autophagy is defective, and apoptosis is induced (42). Thus, we analyzed TRPML1 silencing related effects investigating cell death. An increased number of Annexin V^+^ cells were found in siTRPML1 T98 cells at 24 and 48 h, compared to siGLO control cells (Figure 4). No Annexin V^+^ was detected in siTRPML1 U251 cells. These results suggest the induction of apoptotic cell death in the T98 cell line.

### Transient Receptor Potential Mucolipin 1 Silencing Activates Senescence Program in U251 Cells

**Figure 5:**
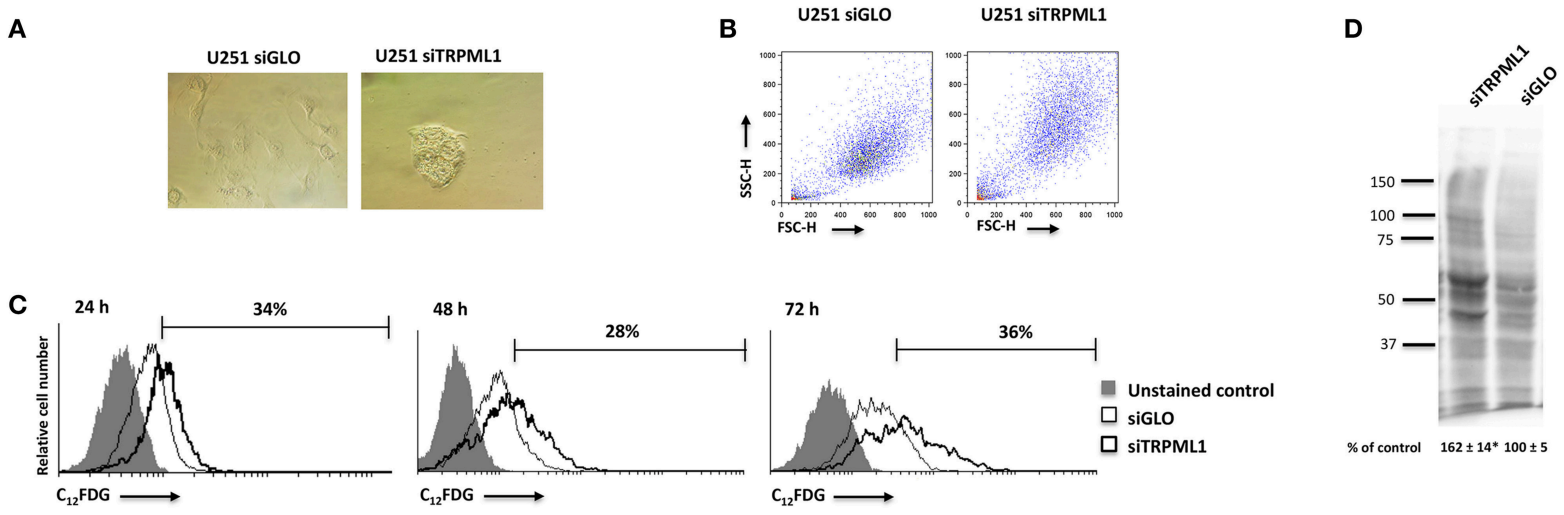
Silencing of transient receptor potential mucolipin 1 (TRPML1) induces senescence in U251 cells. **(A)** Representative image of siGLO and siTRPML1 U251 cells 48 h after silencing. Magnification ×10. **(B)** Dot plots show forward scatter (FSC) and side scatter (SSC) for siGLO and siTRPML1 U251. **(C)** Representative flow cytometric profiles of siGLO and siTRPML1 U251 cells stained with C_12_FDG, a fluorogenic substrate from SA-β-galactosidase before the analysis. Data represent the percentage of positive cells. Gray curve represents unstained cells. **(D)** Carbonyl groups generated by oxidative stress were subjected to DNPH derivatization, and increases in oxidatively modified proteins were detected with an antibody against DNP after size fractionation followed by Western blotting. Quantification of total protein carbonylation was performed from three independent experiments ± SEM. Image is representative of three independent experiments. *p < 0.01 vs. siGLO.

Given the block in G2/M phase of U251 cells, the induction of senescence was evaluated by the morphometric microscope analysis and cytofluorimetric analysis using the scatter parameters and fluorogenic substrate C_12_FDG to detect the SA-β-Galactosidase activity (SA-β-Gal). Briefly, at 48 h after silencing, siTRPML1 and siGLO cells were incubated with 100 nM BAF A1 to induce lysosomal alkalinization at pH 6 and then stained with C_12_FDG. The C_12_-fuorescein signal was measured on the FL-1 detector, and the β-galactosidase activity was quantified. An enlarged cell phenotype was identified by the optic microscope observation and cytofluorimetric analysis, suggesting a senescent phenotype: increased volume of cells and increased values of side and forward scatter (SSC/FSC), indications of increased size and granulosity, were evidenced in siTRPML1 cells compared to siGLO U251 cells after 48 h (Figures 5A,B). Moreover, about 34% of siTRPML1 U251 cells were SA-β-Gal^+^, compared to siGLO cells, already at 24 h, and the senescent phenotype was constant up to 72-h post-transfection (Figure 5C). No SA-β-Gal^+^ cells were found over time in siTRPML1 T98 cells (data not shown).

Given that the phenotypic feature of oxidative stress-induced senescence is the accumulation of carbonylated protein of the Oxi-proteome system (43, 44), we analyzed the amount of this kind of oxidative modification by the OxyBlot^*TM*^ Protein Oxidation Kit (sensibility: <5 femtomoles of carbonyl residue; Merck Millipore, Burlington, MA, USA) and the Western blot analysis after derivatizing carbonyls groups moieties with 2,4-Dinitrophenylhydrazine (DNPH) (Figure 5D). The validation of this technique was performed by omitting the DNPH treatment, the anti-DNP antibody, or the secondary anti-rabbit IgG antibody (not shown). A higher intensity of carbonyl staining was evidenced in siTRPML1 than siGLO U251 cells (Figure 5D).

### Cathepsin B Accumulation and Release in siTRPML1 T98 and U251 Cells

**Figure 6:**
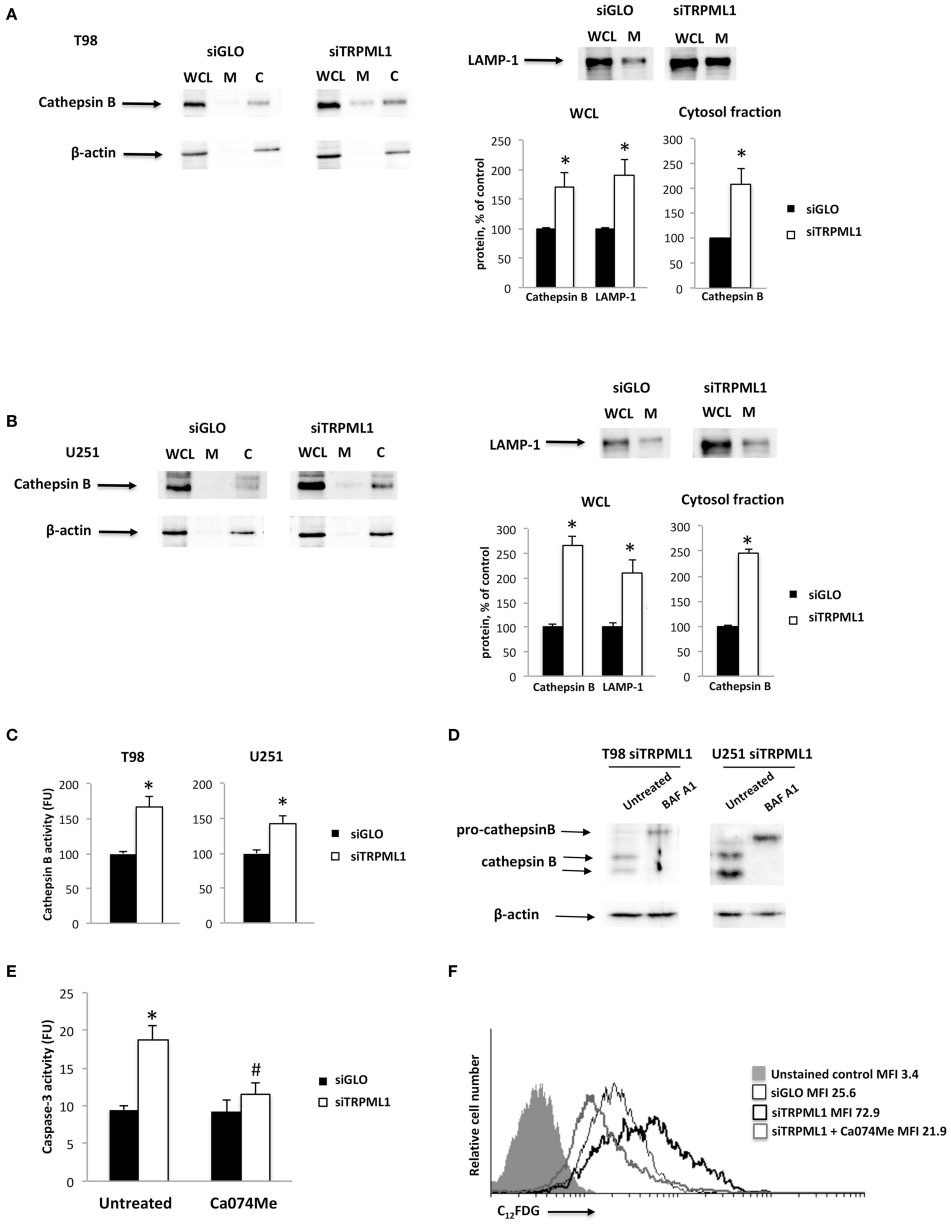
Transient receptor potential mucolipin 1 (TRPML1) silencing induces cathepsin B activation. **(A)** Whole cell lysate (WCL), membrane (M), and cytosolic **(C)** fractions were isolated from siGLO and siTRPML1 T98 cells. **(B)** WCL, M, and C fractions were isolated from siGLO and siTRPML1 T98 cells. Cathepsin B and LAMP-1 levels were measured in WCL by Western blot and normalized to β-actin. Cathepsin B levels were measured in C fraction by Western blot and normalized to β-actin. Densitometric data shown are expressed as mean ± SE of three separate experiments. *p < 0.05. **(C)** Cathepsin B activity was measured in siGLO and siTRPML1 T98 and U251 cells. *p < 0.05. **(D)** siTRPML1 T98 and U251 cells were treated with the inhibitor BAF A1 (25 nM) for 48 h. The Western blot analysis of pro-cathepsin B and mature cathepsin B protein levels was performed. Blots are representative of one of three separate experiments. β-actin was used as loading control. **(E)** Caspase-3 activity was measured in siGLO, and siTRPML1 T98 cells treated or not with the cathepsin inhibitor Ca074Me. FU, fluorescence units. *p < 0.001vs. untreated siGLO, ^#^*p* < 0.05 Ca074Me-treated siTRPML1 vs. Ca074Me-treated siGLO cells. **(F)** Representative flow cytometric profiles of siTRPML1 U251 cells treated or not with Ca074Me and then stained with C12FDG. Gray curve represents unstained cells. MFI, mean fluorescence intensity.

Cathepsin B is a lysosomal protease involved in autophagy and apoptotic cell death (45, 46). Moreover, cathepsin B-dependent apoptosis has been reported in TRPML1 knock-out HeLa cells by Colletti et al. (47). We determined the effects of TRPML1 knock-down on cathepsin B levels by the Western blot analysis at 48-h post-transfection. A marked increase of mature cathepsin B levels was evidenced in silenced T98 and U251 whole cell lysates (WCL) (two-fold) (Figures 6A,B). Cathepsin B is released in response to cellular stress in its active form in the cytosol, where it also promotes the activation of caspases (47). In our model, cathepsin B localization was established using a cytosolic/membrane fractionation protocol. β-actin was used to identify the cytosolic fraction. Immunoblots showed that the 35-kDa mature form of cathepsin B levels was increased both in cytosol fraction in silenced compared to siGLO T98 and U251 cells (Figures 6A,B). In addition, the levels of lysosomal marker LAMP-1 were analyzed showing an increase consistent with previous studies (23, 47, 48).

The significance of cathepsin B upregulation in siTRPML1 cells was evaluated using the fluorogenic peptide Z-Arg-Arg-AMC at a final concentration of 50 μM. The cathepsin B activity was upregulated in both siTRPML1 T98 and U251 cells, compared to siGLO cells (Figure 6C). Moreover, to further evaluate the specificity of siTRPML1 on the cathepsin B activity, the cathepsin D activity was also measured. No cathepsin D activity was evidenced in siTRPML1 cells compared to siGLO T98 and U251 cells (data not shown). To confirm that changes we observed in siTRPML1 T98 and U251 cells were specific and not caused by general endocytic inhibition, siTRPML1 T98 and U251 cells were treated for 48 h with 25 μM of BAF A1, an inhibitor of the lysosomal H^+^ ATPase pump that blocks the acidification of lysosome (49). In agreement with the requirement of active lysosome for pro-cathepsin B processing to mature cathepsin B (50), the BAF A1 treatment resulted in a buildup of the 43-kDa pro-cathepsin B and disappearance of mature forms (Figure 6D).

These data suggest a specific TRPML1-mediated effect on cathepsin B release and activation. The cathepsin B release into the cytosol has been shown to cleave pro-caspase-3 and to induce apoptosis (45–47). Thus, to assess if the apoptosis detected in siTRPML1 T98 cells was cathepsin B-dependent, 24 h of pretreatment with the cell permeable cathepsin B inhibitor Ca074Me (5 μM) was performed on siTRPML1 and siGLO T98 cells. The caspase-3 activity was measured using the fluorogenic substrate Ac-Asp-Glu-Val-Asp-AMC at 5 μM final concentration. Increased caspase-3 activity (two-fold increase) was evidenced in siTRPML1 T98 cells at 48 h, and Ca074Me completely reversed this effect (Figure 6E). Ca074Me alone did not alter the caspase-3 activity in siGLO cells.

Furthermore, cytosolic cathepsin B is also implicated in senescence (51). Hence, to evaluate the role of cathepsin B in a senescent program, we performed experiments on siTRPML1 and siGLO U251 cells using pretreatment for 24 h with Ca074Me (5 μM). The cathepsin B inhibitor completely reversed the increase of SA-β-Gal^+^ cells registered in siTRPML1 cells (Figure 6F). Ca074Me alone did not alter the percentage of SA-β-Gal^+^ cells in siGLO cells.

### Transient Receptor Potential Mucolipin 1 Knock-Down Increases the Invasion Capability of T98 and U251 Cells

**Figure 7:**
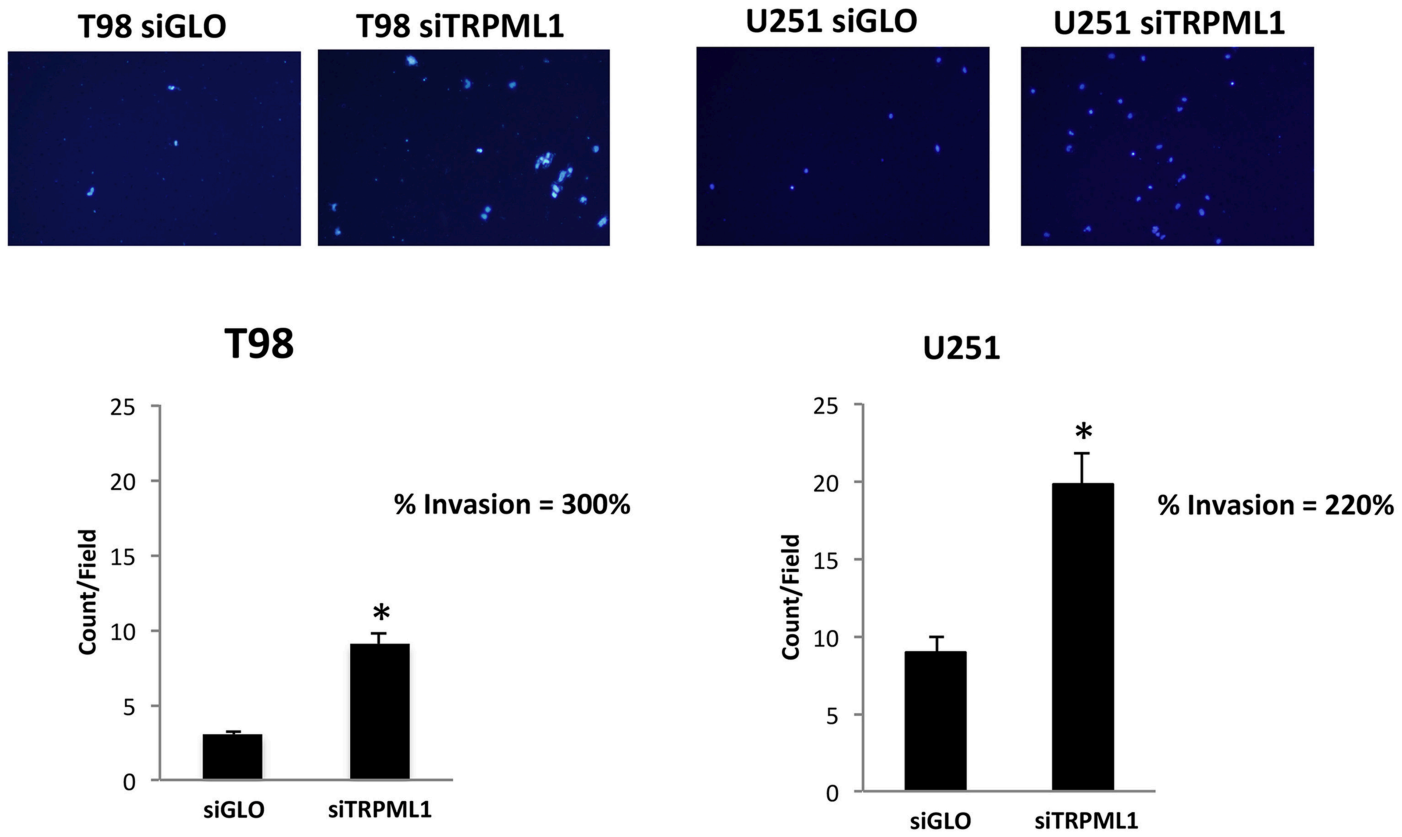
Transient receptor potential mucolipin 1 (TRPML1) silencing increases invasion in glioma cell lines. Representative images showing DAPI fluorescence after 24 h culture in Transwell chambers (×10 magnification). The invading cells were counted in 10 randomly chosen microscopic fields per transwell. Each sample was run in triplicate, and three independent experiments were performed. Bars represent the quantification of invaded cells in each field. Error bars represent ±SE. *p < 0.01.

The effect of TRPML1 on glioblastoma cell line malignancy was investigated *via in vitro* analysis of cell invasion using a Transwell assay. siTRPML1 transfected and siGLO control cells were resuspended in the serum-free medium and added to the upper chamber of each well. A medium with 10% FBS used as a chemoattractant was added to the lower chamber of each well. After 24 h, non-migrating cells were removed. Cells that had migrated to the lower surface of the membrane were fixed with methanol and stained with DAPI. Transfection with siTRPML1 significantly increased invasion in both cell lines (three-fold in T98 and two-fold in U251) when compared with control siRNA transfected cells (Figure 7). This indicates that a negative correlation exists between the TRPML1 expression levels and the motility of glioma cancer cells.

### Transient Receptor Potential Mucolipin 1 Knock-Down Effects in Primary Glioblastoma Cells

**Figure 8:**
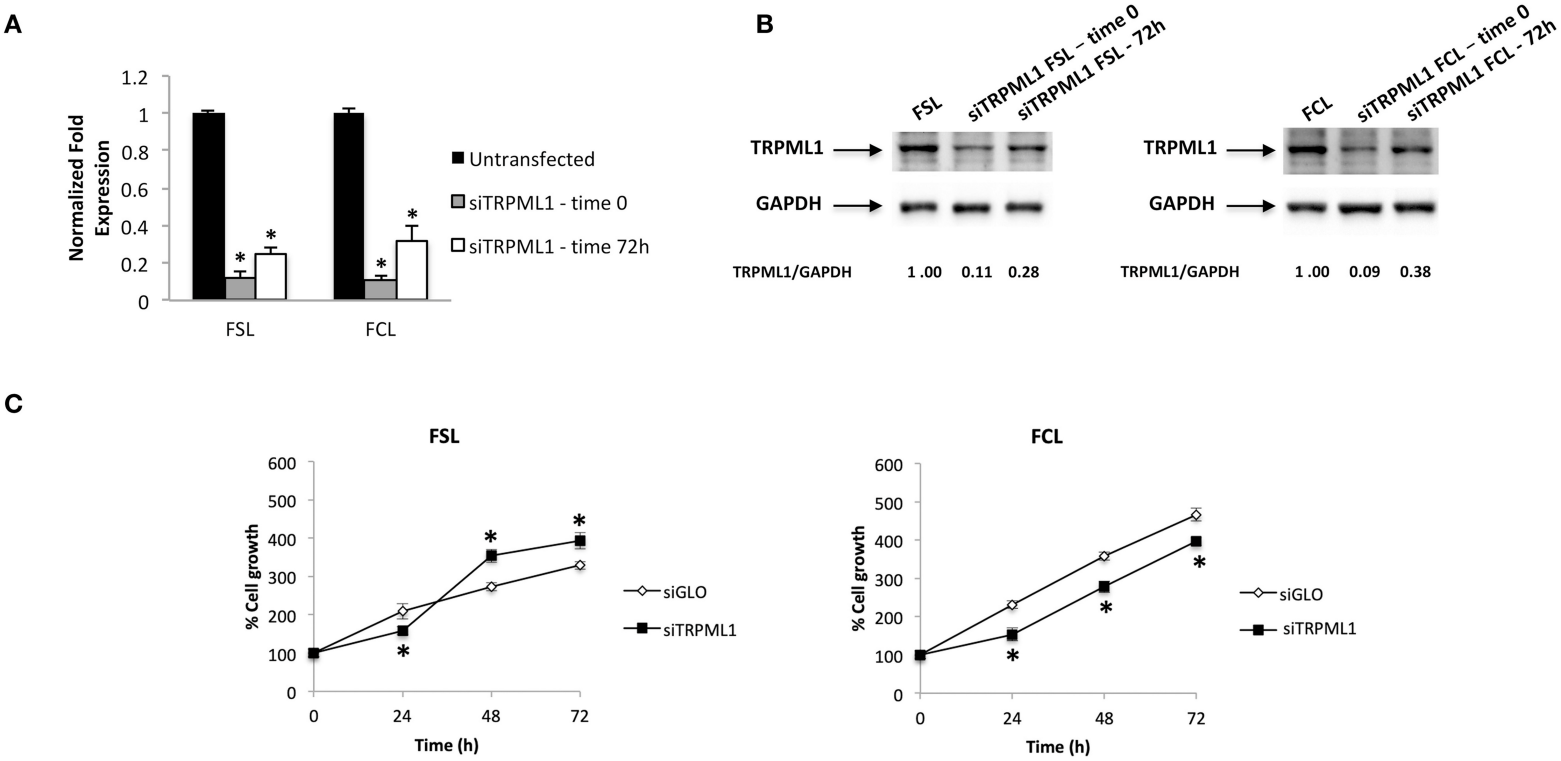
Transient receptor potential mucolipin 1 (TRPML1) silencing affects viability of patient-derived cells. **(A)** The relative TRPML1 mRNA expression in untransfected and siTRPML1 FSL and FCL glioma cell lines after 72 h of silencing (time 0) and after 72-h post-transfection was evaluated by the quantitative reverse transcription (qRT)-PCR. TRPML1 mRNA levels were normalized for glyceraldehyde-3-phosphate dehydrogenase (GAPDH) expression. Data are expressed as mean ± SD; *p < 0.001. **(B)** Western blot analysis of TRPML1 protein levels. Blots are representative of one of three separate experiments. TRPML1 densitometry values were normalized to GAPDH used as loading control. The TRPML1 protein levels of silenced samples were determined with respect to TRPML1 levels of untransfected cells. **(C)** Cell viability was evaluated by the MTT assay in siGLO control and in siTRPML1 FSL and FCL glioblastoma cells for up to 72 h. Data shown are expressed as mean ± SE of three separate experiments. *p < 0.01 siTRPML1 vs. siGLO.

**Figure 9:**
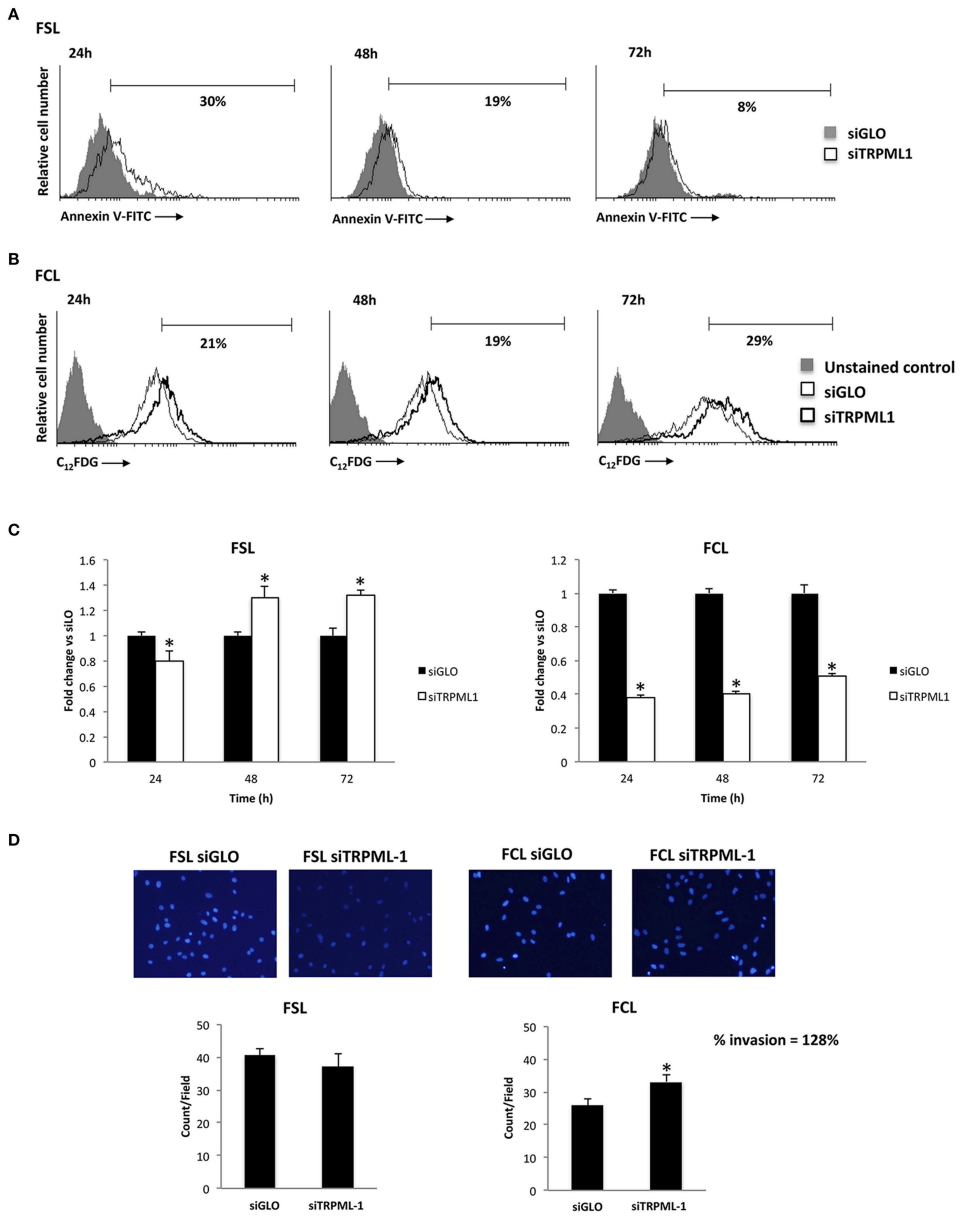
Effects of transient receptor potential mucolipin 1 (TRPML1) silencing on patient-derived cells. **(A)** Annexin V staining was analyzed by flow cytometry in siGLO and siTRPML1 FSL cells for up to 72-h post-silencing. Percentages represent the positive cells. **(B)** Representative flow cytometric profiles of siGLO and siTRPML1 FCL cells stained with C_12_FDG. Data represent the percentage of positive cells. Gray curve represents unstained cells. **(C)** Fold change difference in cell proliferation of siTRPML1 cells was compared with siGLO control cells. *p < 0.05 vs. siGLO. **(D)** Representative images showing DAPI fluorescence after 24-h culture in Transwell chambers (×10 magnification). The invading cells were counted in 10 randomly chosen microscopic fields per transwell. Each sample was run in triplicate, and three independent experiments were performed. Bars represent the quantification of invaded cells in each field. Error bars represent ±SE. *p < 0.05.

Primary glioblastoma cells were transiently transfected with negative transfection control (siGLO) or siRNA against TRPML1. The successful knock-down was validated by the qRT-PCR and Western blot analysis (Figures 8A,B). The transfection efficiency was observed to be in the range of 90% based on GAPDH abundance after 72 h of transfection, called time 0 (Figures 8A,B), and in the range of 60–70% at 72-h post-transfection (Figures 8A,B). Compared to siGLO control cells, the MTT assay evidenced a decrease in cell viability in the first 24 h in siTRPML1 FSL cells, whereas a persistent low percentage of viable cells has been detected in siTRPML1 FCL cells (Figure 8C). In subsequent experiments, Annexin V^+^ siTRPML1 FSL cells have been detected at 24-h post-silencing followed by an increase in BrdU^+^ cells after 48 and 72 h (Figures 9A,C). The other primary cell line FCL was characterized by the activation of senescent program up to 72 h supported by lasting proliferation arrest (Figures 9B,C). Only siTRPML1 FCL cells eventually also acquired invading phenotype as documented by the invasion assay (Figure 9D).

## Discussion

It has been proven in scientific literature that TRPML1 works in controlling cellular homeostasis (23, 24, 49, 50). In addition, the involvement of TRPML1 in cancer has been shown in another study (51), and the present results further strengthen its relevance. We exploited TRPML1 knock-down T98 and U251 cell line models and FSL and FCL patient-derived primary cells.

The silencing of TRPML1 leads to a reduced ERK activation and cell viability; the effect on viability is sustained in siTRPML1 U251 cells, whereas an increased proliferation is observed at 72 h in T98 siTRPML1 cells. In addition, silencing of TRPML1 in T98 and U251 cells induces an oxidative stress response through the RNS generation. It has already been demonstrated that an increase of ROS levels directly activates TRPML1 to induce lysosome Ca^2+^ release, triggering autophagy and/or apoptosis in fibroblast models (23, 49, 52). Pharmacological or genetic knock-down of TRPML1 induces oxidative stress and free radical generations in retinal pigmented epithelial cells (24). Even though the role of TRPML1 as ROS sensor has been demonstrated (53, 54), the consequences of TRPML1 downregulation in glioma cells are still unknown. In agreement with Coblentz et al. data, likely due to ROS levels below the resolution level of DCFDA, the fluorescent ROS sensitive dye used in the cytofluorimetric analysis, we were unable to detect the ROS production in siTRPML1 T98 and U251 cells at any time after transfection (27). However, increased NO generation was detected. Autophagy is a process activated by different stimuli, such as oxidative stress (55) that can interfere with cell fate favoring survival or contributing to cell death. Interestingly, ROS/RNS through TRPML1 activation can induce autophagy and lysosome biogenesis with the consequence of oxidative stress mitigation and promotion of cell survival (26); however, when suprathreshold RNS/ROS levels are reached, it causes lysosome dysfunction, autophagic failure, and apoptosis (26, 56, 57). In this regard, we found that TRPML1 knock-down through high NO production induces defective autophagy in T98 cells, whereas autophagic survival, characterized by an increase of LC3-II and a reduction of p62 levels, was evidenced in siTRPML1 U251 cells, compared to siGLO cells. Moreover, in siTRPML1 T98 cells, the NO generation and defective autophagy result in promoting apoptosis, as evidenced by an increase of Annexin V^+^ cells and caspase-3 activation. This reported apoptosis was cathepsin B-dependent. Evidence was provided in this respect by the ability of NO donor, S-nitroso-N-acetylpenicillamine to induce caspase-dependent apoptosis in glioma cells (58).

Similar to the data reported in siTRPML1 HeLa cells by Colletti et al. (47), knock-down of TRPML1 increased levels of mature cathepsin B (35 kDa) protein both in total cell lysate and in cytosol fraction of siTRPML1, compared to siGLO T98 and U251 cells. This increase is supported by the higher cathepsin B specific activity in both siTRPML1 glioma cell lines, suggesting that it takes part in the activation of caspases leading to apoptotic cell death (45, 46). Actually, the cathepsin B inhibitor, Ca074Me, completely reversed the increase of caspase-3 activity induced in T98 cells by TRPML1 silencing (47). The accumulation of pro-cathepsin B (43 kDa) in BAF A1-treated siTRPML1 T98 and U251 cells eventually confirmed that the increased levels of mature cathepsin B are TRPML1 specific and not due to general endocytic disruption.

Autophagy can block apoptosis (59) but can be also permissive to senescence entry in cells resistant to apoptosis (55, 59). Autophagic-dependent G2/M cell cycle phase arrest upon intracellular free radical generation was reported in esophageal cancer cells (41). Similarly, in siTRPML1 U251 cells, we demonstrated that the autophagy induction and the accumulation of cells in G2/M phase promoted cellular senescence as evidenced by increased SA-β-Gal^+^ cells, compared to siGLO cells. Cathepsin B has also been proposed to play a positive regulatory role in senescence (60). Herein, we demonstrated that cellular senescence induced in U251 cells by TRPML1 silencing is cathepsin B-dependent as demonstrated by using the cathepsin B inhibitor, Ca074Me. It completely abrogated the increase in SA-β-Gal^+^ cells induced by siTRPML1 in U251 cells. Intracellular lysosomal cathepsin B has been demonstrated to induce cellular senescence also in endothelial progenitor cells, where the mature form released in the cytosol clears sirtuin-1, which mediates premature senescence under stress conditions (51). Moreover, Western blot developed with the OxyBlot assay, which has a high sensitivity to carbonyl residue, showed increased levels of oxidized proteins 48 h after transfection in senescent siTRPML1 U251 cells. The OxyBlot kit detects the carbonyl group residues, introduced in a site-specific manner into proteins by oxidative reactions with oxides of nitrous or by metal catalyzed oxidation. The selected cellular proteins that are building up as carbonylated proteins constitute the “Oxi-proteome.” These oxidized proteins comprise components of lysosomal translocation complex, chaperones required for substrate uptake and lysosomal membrane proteins, and energy and redox mitochondrial proteins (61). Our data demonstrated that the TRPML1 silencing induced an accumulation of carbonylated proteins, in agreement with previous studies regarding MLIV (22, 24, 25). TRPML1 mutant fibroblasts from patients with MLIV (22) showing oxidative stress increased the amount of oxidized protein compared to healthy fibroblasts (24, 25). Oxidative carbonylation during oxidative stress has also been found to induce premature senescence in WI-38 human fibroblasts (44).

The most relevant result obtained in this study regards the long-term effects of TRPML1 silencing. Interestingly, the analysis of siTRPML1 cells at 72-h post-transfection highlights the related advantage of siTRPML1 T98 cells resistant to apoptosis that begin to proliferate at a high rate. Instead, the proliferative block and the senescent phenotype in siTRPML1 U251 cells suggest an irreversible senescence. Our previous work demonstrated that low/absent TRPML1 expression is strongly correlated with short survival in patients with glioblastoma, suggesting that the reduction of TRPML1 mRNA expression represents a negative prognostic factor (33). Accordingly, in T98 cells nitrous stress signal induced by TRPML1 knock-down selected an apoptotic resistant, proliferating glioma cell population that quickly grew and overcame the proliferation of siGLO control cells. In siTRPML1 U251 cells, carbonylation of oxidized proteins, through autophagy induction and G2/M arrest, promoted cell senescence. Although senescence arrests cell proliferation and can be considered an obstacle to tumorigenesis given that it is commonly observed in premalignant tumors, several studies detected senescent cells in B-cell lymphoma, lung, breast, colorectal, and thyroid cancers (62–64), and this presence can have a negative impact on patient prognosis. Kim et al. demonstrated that senescent cells are actively involved in the progression of thyroid cancer (65). Indeed, senescent tumor cells exhibit high invasion ability and have an improved survival *via* CXCL12/CXCR4 signaling. Our results demonstrated that T98 proliferating cells and U251 senescent cells show an enhanced ability to invade as a consequence of long-term TRPML1 silencing. In addition, inflammatory senescence-associated secretory phenotype (SASP) factors secreted by senescent cells can promote various aspects of tumorigenesis and immune-mediated clearance (66). Moreover, it was reported that tumor-produced NO has a cancer-promoting role (67, 68). Indeed, the treatment with an NO donor induces motility of glioma cell lines (69), whereas low NO levels due to the inducible NO synthase (iNOS) inhibition block invasiveness and tumor growth *in vivo* (70). In addition, iNOS can promote cancer migration (71, 72), and its expression is negatively correlated with survival in human patients with glioma (73). The different responses of T98 and U251 cells may be related to different susceptibility of glioma cell lines to the TRPML1-mediated effects. The higher levels of RNS produced in T98 cells compared to U251 cells and the increased sensitivity of T98 cells to TRPML1 cytotoxic effects compared to U251 cells together with previously published data in glioma (33) support this hypothesis.

Similar to classic glioblastoma cell lines, TRPML1 silencing in patient-derived primary cultures significantly affected the phenotype of cells. Like T98 cells, but with different kinetics, siTRPML1 FSL cells underwent apoptosis at 24-h post-transfection, whereas an increased proliferation was evidenced at later time points. In addition, cellular senescence and invading ability were induced by TRPML1 silencing in FCL primary glioblastoma cells.

Taken together, all these data suggest that the TRPML1 channel plays a key role in the development and progression of high-grade glioma. The transient silenced models demonstrated that the loss of TRPML1 expression enables a more aggressive behavior to be established, which can promote proliferation and/or invasion. Thus, these findings suggest that a long-term downregulation of TRPML1 expression facilitates the acquisition of malignant features responsible for rapid invasion.

Furthermore, glioblastoma is a tumor characterized by high levels of heterogeneity, one of the main causes of resistance to conventional treatments (8). For this reason, there is a growing interest in identifying new molecular actors to consider new treatment options for glioblastoma. Given that the molecular heterogeneity among our different glioblastoma cell lines mirrors the intercellular heterogeneity in cancer, our data suggest a central role for TRPML1 in tumorigenesis and further strengthens the hypothesis of TRPML1 downregulation as a negative prognostic factor in glioblastoma. Among the emerging and underexplored targets to be taken into account, TRPML1 can be included.

## Conclusion

Glioblastoma is the most fatal primary brain tumor. The heterogeneous nature of this cancer within and across tumors underscores the difficulty of developing efficacious treatments and provides challenges for both understanding the disease and stratifying patients for treatment.

Gene expression and survival data analysis of patients with glioblastoma identified low TRPML1 expression as a negative prognostic marker (33). In this study, we demonstrated that sustained TRPML1 downregulation could result in a survival advantage or in the acquisition of invasion ability in cancer cells.

These findings offer scientists a better understanding of TRPML1 ability in tumors and clear implications for therapy options.

## Data Availability Statement

The raw data supporting the conclusions of this article will be made available by the authors, without undue reservation.

## Author Contributions

GS designed and wrote the paper. MM performed experiments, analyzed data, and co-wrote the paper. CA performed flow cytometry analysis. AA participated in the experiments with primary patient-derived cells. AE performed fluorimetric analysis. CA, MN, OM, FM, and MS contributed in writing and revision. All authors read and approved the paper for the publication.

## Conflict of Interest

The authors declare that the research was conducted in the absence of any commercial or financial relationships that could be construed as apotential conflict of interest.

## Supplementary Material

Supplementary Figure 1 To analyze reactive oxygen species (ROS) production siGLO and siTRPML1, T98 and U251 cells were stained with dichlorodihydrofluorescein diacetate (DCFDA) before the flow cytometric analysis. Histograms are representative of one of three separate experiments. MFI, mean fluorescence intensity.
