# Supplementary figures and images for: Knock-Down of Mucolipin 1 Channel Promotes Tumor Progression and Invasion in Human Glioblastoma Cell Lines
Source: Front Oncol. 2021 Apr 19;11:578928. doi: 10.3389/fonc.2021.578928 (PMC8092188; doi:10.3389/fonc.2021.578928)

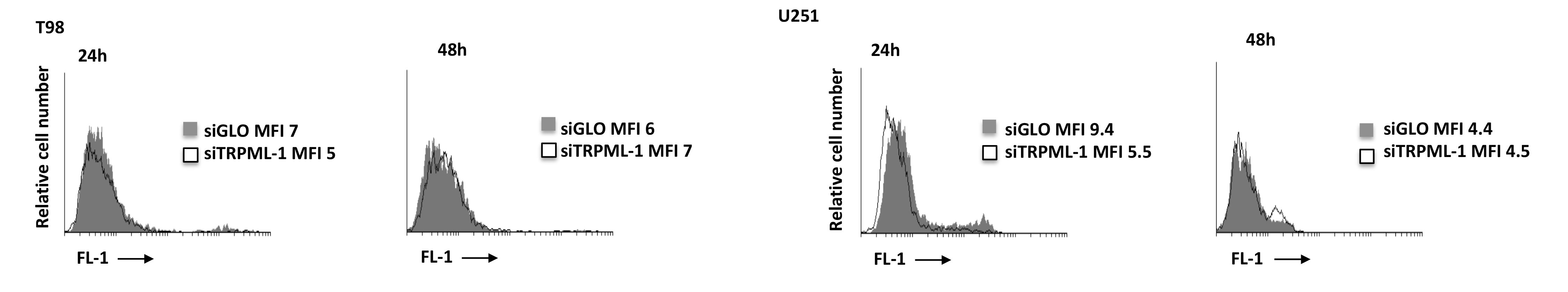

Supplement: Supplementary Figure 1 — To analyze reactive oxygen species (ROS) production siGLO and siTRPML1, T98 and U251 cells were stained with dichlorodihydrofluorescein diacetate (DCFDA) before the flow cytometric analysis. Histograms are representative of one of three separate experiments. MFI, mean fluorescence intensity. [file Image_1.TIF]
